# Tissue alkaline phosphatase activity and expression in an experimental infant swine model of cardiopulmonary bypass with deep hypothermic circulatory arrest

**DOI:** 10.1186/s12950-020-00256-2

**Published:** 2020-08-12

**Authors:** Ludmila Khailova, Justin Robison, James Jaggers, Richard Ing, Scott Lawson, Amy Treece, Danielle Soranno, Suzanne Osorio Lujan, Jesse A. Davidson

**Affiliations:** 1 Department of Pediatrics, University of Colorado, 13123 East 16th Ave, Box 100, Aurora, CO 80045 USA; 2 Department of Surgery, University of Colorado, Aurora, CO USA; 3 Department of Anesthesiology, University of Colorado, Aurora, CO USA; 4 Children’s Hospital Colorado, Heart Institute, Aurora, CO USA; 5 Department of Pathology, University of Colorado, Aurora, CO USA

**Keywords:** Cardiac surgery, Organ injury, Inflammation, Endotoxin, Acute kidney injury, Acute lung injury, Pediatric, Congenital heart disease, Therapy, Neonate

## Abstract

**Background:**

Infant cardiac surgery with cardiopulmonary bypass results in decreased circulating alkaline phosphatase that is associated with poor postoperative outcomes. Bovine intestinal alkaline phosphatase infusion represents a novel therapy for post-cardiac surgery organ injury. However, the effects of cardiopulmonary bypass and bovine-intestinal alkaline phosphatase infusion on tissue-level alkaline phosphatase activity/expression are unknown.

**Methods:**

Infant pigs (*n* = 20) underwent cardiopulmonary bypass with deep hypothermic circulatory arrest followed by four hours of intensive care. Seven control animals underwent mechanical ventilation only. Cardiopulmonary bypass/deep hypothermic circulatory arrest animals were given escalating doses of bovine intestinal alkaline phosphatase infusion (0-25 U/kg/hr.; *n* = 5/dose). Kidney, liver, ileum, jejunum, colon, heart and lung were collected for measurement of tissue alkaline phosphatase activity and mRNA.

**Results:**

Tissue alkaline phosphatase activity varied significantly across organs with the highest levels found in the kidney and small intestine. Cardiopulmonary bypass with deep hypothermic circulatory arrest resulted in decreased kidney alkaline phosphatase activity and increased lung alkaline phosphatase activity, with no significant changes in the other organs. Alkaline phosphatase mRNA expression was increased in both the lung and the ileum. The highest dose of bovine intestinal alkaline phosphatase resulted in increased kidney and liver tissue alkaline phosphatase activity.

**Conclusions:**

Changes in alkaline phosphatase activity after cardiopulmonary bypass with deep hypothermic circulatory arrest and bovine intestinal alkaline phosphatase delivery are tissue specific. Kidneys, lung, and ileal alkaline phosphatase appear most affected by cardiopulmonary bypass with deep hypothermic circulatory arrest and further research is warranted to determine the mechanism and biologic importance of these changes.

## Background

Congenital heart disease (CHD) is a serious birth defect affecting 1 in 120 babies born in the United States (~ 40,000 births) [1]. About 25% of these infants require surgical repair or palliation of critical CHD in the first year of life [2]. Repair frequently involves cardiopulmonary bypass (CPB) with or without additional techniques to maintain a clear operative field such as deep hypothermic circulatory arrest (DHCA) or selective cerebral perfusion [3]. Unfortunately, these techniques are not benign and can lead to severe postoperative complications such as systemic inflammation and multiple organ injury [4–11]. Treatment for postoperative inflammation and organ injury is largely limited to supportive care.

Alkaline phosphatases (AP) are ubiquitous plasma membrane-anchored enzymes, conserved from bacteria to humans, that catalyze the hydrolysis of phosphate groups from different substrates [12, 13]. Four forms of AP have been identified in humans encoded by 4 different genes: tissue-nonspecific (TNAP), intestinal (iAP), placental (pAP) and placental-like (plAP) [13, 14]. TNAP subsequently undergoes post-translational processing, resulting in bone and liver isoforms [15, 16]. TNAP is the dominant circulating isoenzyme and has been successfully used as a biomarker of liver and bone disease [17]; however, its physiological role is not fully understood. Recently, APs have been shown to dephosphorylate and detoxify two key pathologic molecules thought to contribute to inflammation and organ injury after both cardiac surgery and sepsis: bacterial-derived lipopolysaccharide (LPS-endotoxin) [10, 18–25] and extracellular adenine nucleotides (ATP, ADP, and AMP) released during cellular necrosis or apoptosis [26–30]. AP removes one phosphate group from the conserved lipid A moiety of LPS resulting in a monophosphoryl product with reduced toxicity [21, 31]. AP dephosphorylation of extracellular adenine nucleotides results in creation of adenosine, which has multiple beneficial effects including decreased inflammation, decreased platelet aggregation, vasodilation, and decreased ischemia-reperfusion injury [32–37].

In phase 2 clinical trials AP infusion has been shown to improve inflammation and outcomes across several inflammatory diseases including inflammatory bowel disease, sepsis, and ischemia/reperfusion [38–42]. AP may also serve as part of the host-defense system for patients undergoing cardiac surgery. Adult and pediatric patients undergoing cardiac surgery with CPB demonstrate a steep decline in circulating AP activity in the immediate postoperative period [43–45]. Our group has recently shown that lower postoperative AP activity is associated with increased need for vasoactive and inotropic support, increased odds of cardiac arrest, ECMO, or death, and increased biomarker evidence of intestinal and kidney injury [46]. Furthermore, decreased circulating AP resulted in decreased ability to clear endogenous LPS or exogenous AMP in clinically obtained post-CPB serum samples. Clearance could be improved by ex vivo addition of either exogenous bovine intestinal AP (BiAP) or human liver AP [47]. These clinical studies, however, are limited in their ability to comprehensively detail changes in native AP after cardiac surgery with CPB as they examine only circulating AP and are unable to measure tissue-level changes in AP activity or expression. Our group recently demonstrated that kidney AP activity decreases after CPB in a piglet model of infant CPB with DHCA and that decreased levels of renal tissue AP were associated with increased histologic acute kidney injury [48]. High dose infusion of BiAP during CPB increased both serum and renal tissue AP activity with a trend towards reduction in histologic acute kidney injury [48]. It is unclear whether AP expression and activity in other organs exposed to CPB/DHCA would follow a similar pattern or if BiAP could increase AP activity in other at-risk organs.

Organ-specific AP levels may play a role in each organ’s response to ischemia/reperfusion injury. In this work, we utilize our piglet model of infant CPB/DHCA to determine changes in organ-specific total AP activity and TNAP/iAP mRNA induced by CPB/DHCA. We also tested the effects of escalating doses of BiAP infusion on organ-specific total AP activity and TNAP/iAP mRNA production. We hypothesized that CPB/DHCA would result in decreased total AP activity in all organs with variable changes in AP mRNA expression depending on the primary AP isoenzyme produced in each organ. Furthermore, animals receiving BiAP infusion would demonstrate increased total AP activity in all organs.

## Results

### Total AP activity, TNAP and iAP mRNA levels in control animals

**Fig. 1:**
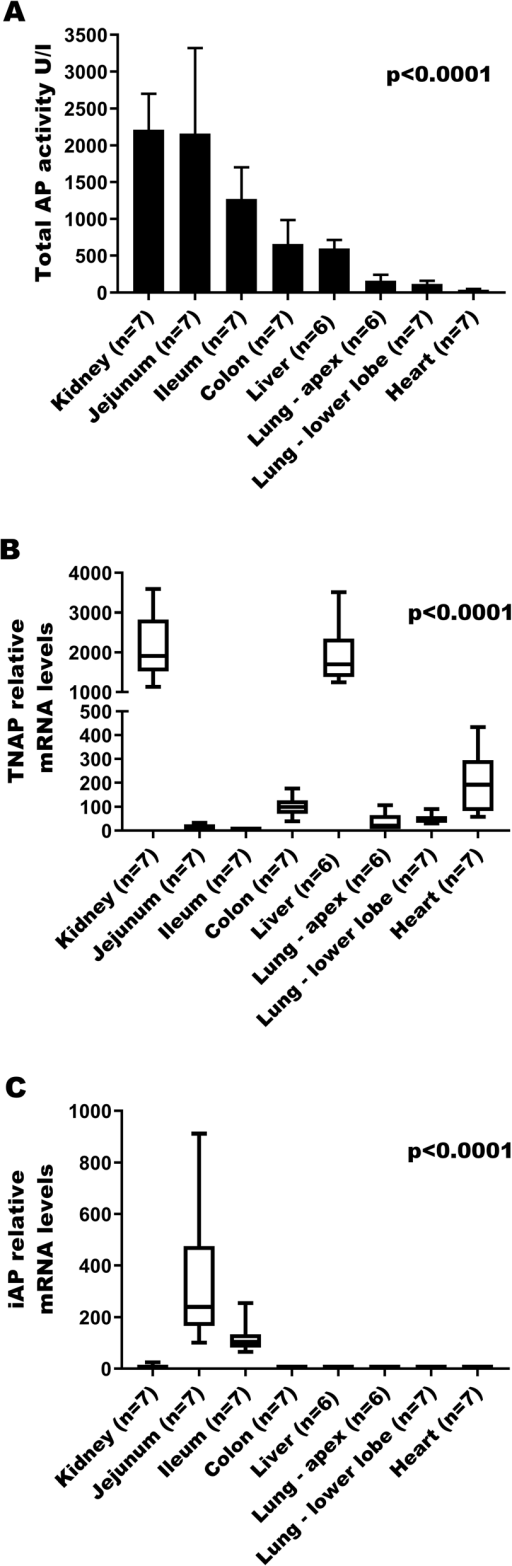
Tissue AP activity and mRNA expression for anesthesia controls. (Legend): Alkaline phosphatase (AP) measured in tissue of anesthesia control animals: **a**) total activity, **b**) relative mRNA levels of tissue non-specific AP (TNAP), and **c**) relative mRNA levels of intestinal AP (iAP) normalized to ileum. Data are expressed as means (SD) [AP activity] or medians with intra-quartile ranges [mRNA levels]. One-way ANOVA or Kruskal-Wallis test were performed as appropriate for the data

We first measured total AP activity, TNAP mRNA expression and iAP mRNA expression in kidneys, intestines, liver, heart, and lungs of anesthesia controls to obtain baseline levels. Total AP activity was well represented by a normal distribution and is reported as means (SD). mRNA expression was not normally distributed and is therefore reported as medians (intra-quartile range). The results revealed statistically significant differences in the distribution of total AP activity across tissues (*p* < 0.0001). Kidney, jejunum and ileum showed the highest total AP activity, followed by moderate levels in liver and colon, and low levels in the lungs (apex and lower lobe) and heart (Fig. 1a). The relative TNAP and iAP mRNA expression results were normalized to mRNA levels in the ileum (organ with the lowest TNAP expression; iAP data were multiplied by 100 to better graphically present data). TNAP mRNA was significantly differentially expressed across the organs assessed (*p* < 0.0001) (Fig. 1b). Kidney and liver had the highest expression, while the lungs (right apex and right lower lobe) and heart expressed low TNAP mRNA levels. The mRNA expression of TNAP in the intestines (jejunum, ileum and colon) was minimal. In contrast, iAP mRNA expression was 100–300 times higher in the jejunum and ileum when compared to the rest of the organs (Fig. 1c).

### Total AP activity change after CPB/DHCA and the effect of BiAP supplementation

**Fig. 2:**
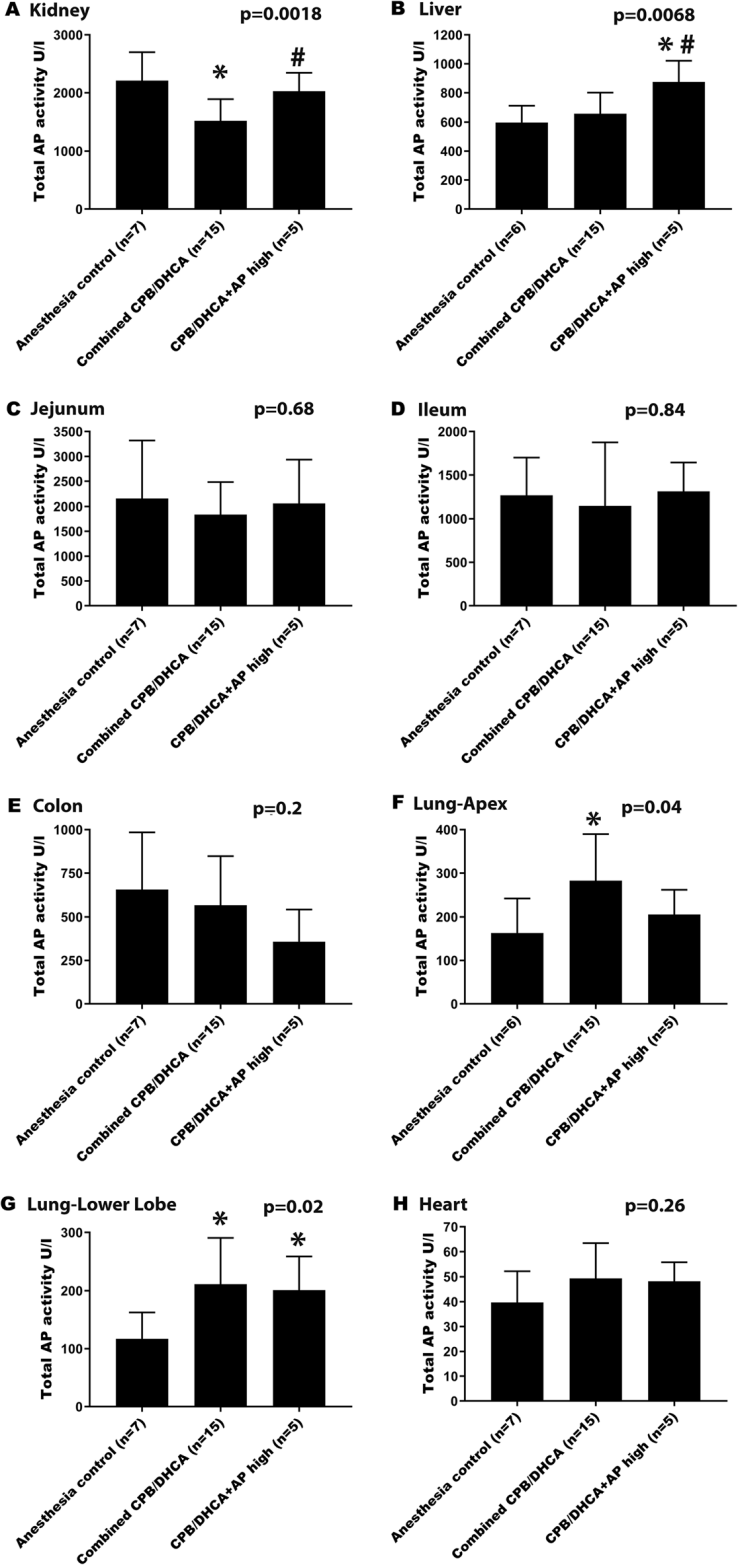
Effects of CPB/DHCA and BiAP infusion on tissue AP activity. (Legend): Comparison of total alkaline phosphatase (AP) activity in tissue of anesthesia controls, CBP/DHCA animals receiving no/low/medium dose BiAP (Combined CPB/DHCA), and CPB/DHCA animals receiving high dose BiAP infusion (CPB/DHCA + AP high). **a** Kidney **b** Liver **c** Jejunum **d** Ileum **e** Colon **f** Lung – apex **g** Lung – lower lobe **h** Heart. Data are expressed as medians with intra-quartile ranges. One-way ANOVA was performed to assess for difference among groups (overall *p*-value). Post-hoc Student’s t-testing was performed to identify differential groups. * = *p* < 0.05 compared to anesthesia controls; # = p < 0.05 compared to combined CPB/DHCA group

Total AP activity in each organ is shown in Table 1 by intervention group. In addition, we previously reported that infusion of low and medium doses of BiAP did not significantly alter the total serum or kidney tissue AP activity. We therefore also analyzed groups based on effective circulating drug levels achieved (Fig. 2), combining CPB/DHCA, CPB/DHCA+AP low and CPB/DHCA+AP medium groups (combined CPB/DHCA) for comparison to the CPB/DHCA+AP high group and the anesthesia controls.

**Table 1:** Comparison of tissue total alkaline phosphatase (AP) activity by intervention group

Tissue	Intervention Group Total AP Activity (U/L) Mean (SD)					***P*** value (ANOVA)	Significant Post-HOC Group Comparisons
	AC (0)	CPB/DHCA (1)	CPB/DHCA + AP Low (2)	CPB/DHCA + AP Medium (3)	CPB/DHCA + AP High (4)		
Kidney	2211 (491)	1628 (472)	1536 (441)	1404 (165)	2028 (318)	**0.01**	0 vs. 1; 0 vs. 2; 0 vs. 3; 3 vs. 4
Liver	598 (115)	642 (161)	725 (100)	609 (161)	875 (146)	**0.02**	0 vs. 4; 1 vs. 4; 3 vs. 4
Jejunum	2160 (1162)	1460 (663)	1760 (477)	2292 (586)	2060 (877)	0.51	
Ileum	1270 (432)	1016 (368)	982 (508)	1450 (1135)	1312 (332)	0.71	
Colon	658 (327)	664 (435)	500 (97)	540 (238)	358 (185)	0.40	
Lung-Apex	163 (79)	263 (153)	305 (108)	276 (82)	206 (57)	0.17	
Lung-Lower Lobe	117 (46)	137 (21)	216 (85)	280 (38)	201 (57)	**0.0003**	0 vs. 2; 0 vs. 3; 0 vs. 4; 1 vs. 2; 1 vs. 3; 3 vs. 4
Heart	40 (12)	47 (15)	51 (19)	50 (10)	48 (8)	0.57	

Group 0 = Anesthesia controls (AC); Group 1 = Animals undergoing CBP/DHCA; Group 2 = Animals undergoing CPB/DHCA with low dose BiAP (3 U/kg bolus followed by 1 U/kg/hr. infusion); Group 3 = Animals undergoing CPB/DHCA with medium dose BiAP (15 U/kg bolus followed by 5 U/kg/hr. infusion); Group 4 = Animals undergoing CPB/DHCA with high dose BiAP (75 U/kg bolus followed by 25 U/kg/hr. infusion). Data are expressed as means (SD). One-way ANOVA was performed followed by post-hoc Student’s t-tests as appropriate. Bold: significant at *p* < 0.05

As previously published, kidney tissue AP activity was significantly lower in animals undergoing CPB/DHCA compared to anesthesia controls (Table 1) [48]. Total AP activity in the jejunum, ileum, colon, liver and heart did not differ significantly between the anesthesia control and CPB/DHCA groups (Table 1) or the anesthesia control group and the combined CPB/DHCA group (Fig. 2b, c, Dd, e, h). Interestingly, total AP activity in the lung (both upper and lower lobes) trended higher in all CPB/DHCA groups compared to anesthesia controls (Table 1) without a dose-response effect following AP administration. Lung AP activity (upper and lower lobes) was significantly higher in the combined CPB/DHCA group compared to anesthesia controls (Fig. 2f, g). High dose BiAP infusion led to a significant increase in AP activity in kidneys and liver when compared to the combined CPB/DHCA group (Fig. 2a, b) but did not significantly change total AP activity in the jejunum, ileum, colon, lungs or heart (Fig. 2c-g).

### Change in TNAP and iAP mRNA expression after CPB/DHCA

**Fig. 3:**
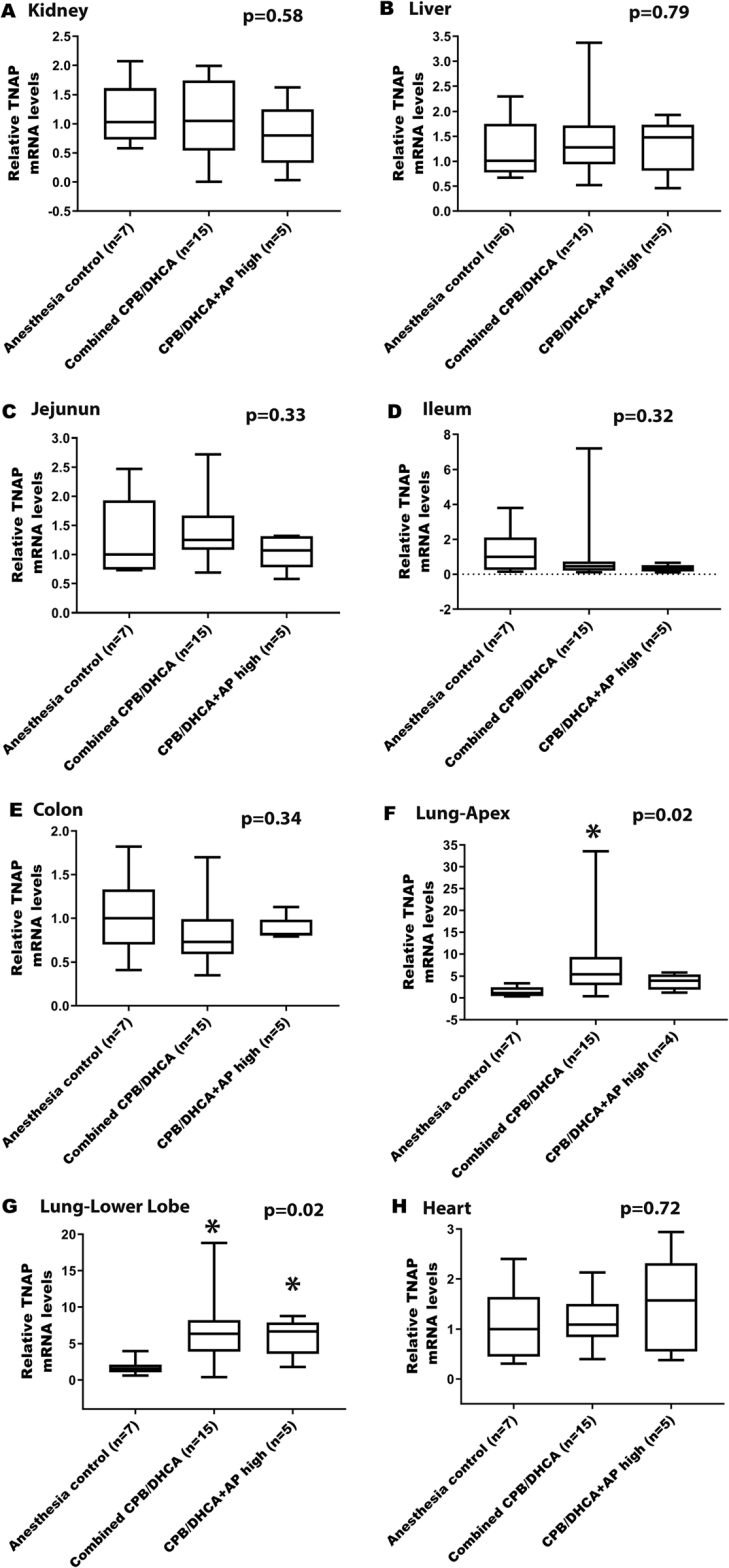
Effects of CPB/DHCA and BiAP infusion on tissue-nonspecific AP mRNA expression. (Legend): Comparison of tissue non-specific alkaline phosphatase (TNAP) mRNA expression in tissue of anesthesia controls, CBP/DHCA animals receiving no/low/medium dose BiAP (Combined CPB/DHCA), and CPB/DHCA animals receiving high dose BiAP infusion (CPB/DHCA + AP high). **a** Kidney **b** Liver **c** Jejunum **d** Ileum **e** Colon **f** Lung – apex **g** Lung – lower lobe **h** Heart. Data are expressed as medians with intra-quartile ranges. Kruskal-Wallis testing was performed to assess for difference among groups (overall *p*-value). Post-hoc Mann-Whitney U-testing was performed to identify differential groups. * = p < 0.05 compared to anesthesia controls

Although total renal AP activity in CPB/DHCA animals was significantly decreased, we did not find any differences in TNAP mRNA expression compared to anesthesia controls, suggesting that decreased AP activity in CPB/DHCA kidneys was secondary to loss or consumption of AP rather than changes in production (Table 2 and Fig. 3a). In contrast, both the apex and lower lobe of the lung showed a significant increase in TNAP mRNA expression in the combined CPB/DHCA group of animals compared to anesthesia controls (Fig. 3f, g). This increase in TNAP expression is likely the primary contributor to increased tissue AP activity in the lungs following exposure to CPB/DHCA. We found no evidence in favor of altered TNAP mRNA expression induced by CPB/DHCA in liver or heart tissue (Fig. 3b, h).

**Table 2:** Comparison of tissue non-specific alkaline phosphatase (TNAP) mRNA expression

Tissue	TNAP Relative mRNA Levels by Intervention Group Median (Intra-Quartile Range)					P value (Kruskal-Wallis)
	AC (0)	CPB/DHCA (1)	CPB/DHCA + AP Low (2)	CPB/DHCA + AP Medium (3)	CPB/DHCA + AP High (4)	
Kidney	1.03 (0.73–1.61)	1.42 (0.80–1.91)	0.65 (0.022–1.51)	1.00 (0.67–1.48)	0.80 (0.33–1.25)	0.51
Liver	1.01 (0.78–1.75)	1.28 (0.83–1.82)	0.94 (0.52–1.71)	1.67 (1.31–2.55)	1.48 (0.81–1.74)	0.42
Jejunum	1.00 (0.74–1.93)	1.35 (1.11–2.21)	1.56 (0.99–1.82)	1.13 (1.07–1.21)	1.07 (0.78–1.32)	0.41
Ileum	1.00 (0.25–2.11)	0.21 (0.15–3.84)	0.74 (0.58–0.81)	0.35 (0.19–0.58)	0.38 (0.16–0.53)	0.22
Colon	1.00 (0.70–1.33)	0.81 (0.69–1.55)	0.73 (0.47–1.20)	0.65 (0.39–0.84)	0.82 (0.80–0.99)	0.29
Lung-Apex	1.12 (0.41–2.47)	6.14 (4.02–11.06)	5.47 (1.65–19.57)	5.46 (1.87–9.61)	3.99 (1.91–5.40)	0.089
Lung-Lower Lobe	1.57 (1.04–2.13)	5.08 (2.44–13.11)	6.36 (4.95–11.76)	6.60 (1.19–9.21)	6.68 (3.62–7.94)	0.067
Heart	1.00 (0.45–1.64)	1.09 (0.64–1.36)	1.43 (0.85–1.53)	0.84 (0.71–1.72)	1.57 (0.55–2.32)	0.89

Group 0 = Anesthesia controls (AC); Group 1 = Animals undergoing CBP/DHCA; Group 2 = Animals undergoing CPB/DHCA with low dose BiAP (3 U/kg bolus followed by 1 U/kg/hr. infusion); Group 3 = Animals undergoing CPB/DHCA with medium dose BiAP (15 U/kg bolus followed by 5 U/kg/hr. infusion); Group 4 = Animals undergoing CPB/DHCA with high dose BiAP (75 U/kg bolus followed by 25 U/kg/hr. infusion). Data are expressed as medians with intra-quartile ranges. Kruskal-Wallis test was performed

**Fig. 4:**
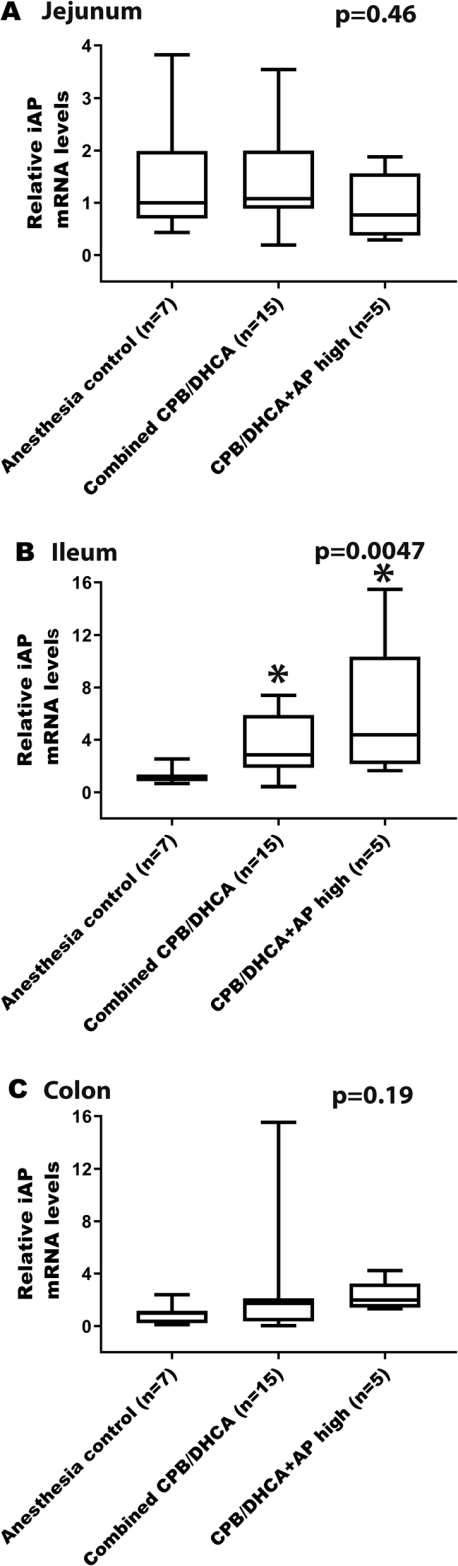
Effects of CPB/DHCA and BiAP infusion on intestinal AP mRNA expression. (Legend): Comparison of intestinal alkaline phosphatase (iAP) mRNA expression in tissue of anesthesia controls (AC), CBP/DHCA animals receiving no/low/medium dose BiAP (Combined CPB/DHCA), and CPB/DHCA animals receiving high dose BiAP infusion (CPB/DHCA + AP high). **a** Jejunum **b** Ileum **c** Colon. Data are expressed as medians with intra-quartile ranges. Kruskal-Wallis testing was performed to assess for difference among groups (overall p-value). Post-hoc Mann-Whitney U-testing was performed to identify differential groups. * = p < 0.05 compared to anesthesia controls

Changes in AP mRNA expression in the intestines were more complex. Exposure to CPB/DHCA did result in increased iAP mRNA expression in the ileum with a similar trend in the colon when compared to the anesthesia controls (Table 3 and Fig. 4b, c). This finding of higher iAP mRNA expression but unchanged-to-lower AP activity in the ileum and colon likely reflect a combination of increased production with ongoing loss/consumption. CPB/DHCA did not alter iAP mRNA expression in the jejunum (Table 3 and Fig. 4a). TNAP mRNA expression was not increased in either the small intestine or the colon (Table 2 and Fig. 3c, d, e).

**Table 3:** Comparison of intestinal alkaline phosphatase (iAP) mRNA expression in intestinal tissue

Tissue	iAP Relative mRNA Levels by Intervention Group Median (Intra-Quartile Range)					P value (Kruskal-Wallis)	Significant Post-HOC Group Comparisons
	AC (0)	CPB/DHCA (1)	CPB/DHCA + AP Low (2)	CPB/DHCA + AP Medium (3)	CPB/DHCA + AP High (4)		
Jejunum	1.00 (0.70–1.99)	1.08 (0.60–2.20)	0.96 (0.79–1.20)	2.00 (1.07–2.85)	0.77 (0.37–1.57)	0.37	
Ileum	1.04 (0.82–1.33)	2.85 (1.14–5.71)	3.06 (2.21–5.14)	2.64 (1.93–6.14)	4.38 (2.15–10.35)	**0.027**	0 vs. 2; 0 vs. 3; 0 vs. 4
Colon	1.00 (0.22–1.18)	1.99 (0.20–8.76)	1.72 (0.87–2.09)	1.02 (0.24–4.55)	1.98 (1.41–3.25)	0.49	

Group 0 = Anesthesia controls (AC); Group 1 = Animals undergoing CBP/DHCA; Group 2 = Animals undergoing CPB/DHCA with low dose BiAP (3 U/kg bolus followed by 1 U/kg/hr. infusion); Group 3 = Animals undergoing CPB/DHCA with medium dose BiAP (15 U/kg bolus followed by 5 U/kg/hr. infusion); Group 4 = Animals undergoing CPB/DHCA with high dose BiAP (75 U/kg bolus followed by 25 U/kg/hr. infusion). Data are expressed as medians with intra-quartile ranges. Kruskal-Wallis test was performed followed by post-hoc non-parametric Mann-Whitney U-test as appropriate. Bold: significant at p < 0.05

### Effect of high dose BiAP infusion on tissue AP activity

The effect of high dose BiAP infusion on tissue AP activity varied across organs. Total AP activity was significantly elevated in the kidneys and liver in the high dose BiAP group (Table 1 and Fig. 1a, b) without an increase in mRNA expression, suggesting successful delivery of BiAP to these organs. No other organ assessed demonstrated a significant increase in tissue AP activity with high dose AP infusion (Table 1 and Fig. 1c-h).

## Discussion

### Key findings

To our knowledge, this study is the first to comprehensively define changes in organ-specific AP activity and expression induced by CPB/DHCA, as well as changes in tissue-level AP activity with the infusion of exogenous BiAP. In control infant pigs not exposed to CPB/DHCA (anesthesia with mechanical ventilation only), the highest total AP activity was found in kidneys, followed by the intestines and liver with lower activity in the lungs and heart. TNAP mRNA was the dominant isoenzyme expressed in the kidneys and liver, while the jejunum and ileum predominantly expressed the intestinal isoenzyme. Exposure to CPB/DHCA resulted in a decrease in kidney AP activity but an increase in lung AP activity, with no significant changes in total AP activity in the other organs assessed. We found no evidence that kidney AP mRNA expression was altered by CPB/DHCA, suggesting that decreased tissue AP activity was due to enzyme loss rather than decreased transcription. In contrast, CPB/DHCA resulted in increased expression of TNAP mRNA in the lung, likely leading to the increased tissue AP activity. The ileum demonstrated a mixed picture, with increased expression of its predominant AP isoenzyme (iAP) following exposure to CPB/DHCA, but no change in total tissue AP activity, suggesting a combination of increased production with ongoing AP loss. Treatment of the piglets undergoing CPB/DHCA with the highest dose of BiAP resulted in increased total AP activity in kidney and liver only. BiAP infusion did not appear to significantly alter AP mRNA expression in any organ.

### Organ-specific AP activity and expression in healthy controls

AP is used routinely as a biomarker for liver and bone disease and most clinicians are familiar with these two sources of AP. It is less commonly appreciated that AP is a ubiquitous enzyme that is conserved from bacteria to humans and is present in most organs, including kidney, intestines, and lung in addition to liver and bone [16, 49–53]. Relatively little is known regarding the biology of AP at the tissue level or its organ-specific role/response to disease. In this study, we demonstrate that the highest tissue AP activity is found not in the liver but instead in the kidneys and small intestine (bone was not tested), with intermediate activity in the liver and colon and lower activity in the thoracic organs. These findings validate early studies in large animals, [54, 55] which similarly found the highest levels of AP activity in the kidneys and intestines. As expected, the specific isoenzyme expressed differed between these organs, with the ileum and jejunum largely expressing the intestinal isoform and the kidneys expressing the tissue nonspecific isoform. This expression pattern is also consistent with prior studies in small and large animals as well as human studies and supports the utility of pigs as a large animal model of AP biology.

The biologic role for high AP expression in the kidneys and intestines in healthy animals has not been fully elucidated. In both of these organs AP localizes primarily to the epithelial surfaces: the proximal tubules of the kidneys [56] and the microvilli along the apical surface of intestinal enterocytes [57, 58]. IAP is expressed throughout the intestines, with the highest levels in the duodenum and lower levels in the colon [59]. High concentrations of iAP are found in apical vesicles that can secrete functional iAP into the intestinal lumen [59, 60]. Recent studies of AP in the intestines point towards a protective role against LPS and other microbacterial products, consistent with their brush border localization [16, 59, 61, 62]. AP may also have a role in absorption of nutrients from the intestinal lumen [16, 59, 61, 63, 64]. The biologic role of epithelial AP in healthy kidneys is less clear [65]. TNAP is the predominant isoform and is located in the S1, S2, and S3 segments of the proximal tubule with a small amount of iAP present in the S3 segment as well [65–67]. Preclinical data suggest that AP contributes to maintenance of inorganic phosphate homeostasis [63] and may lead to adenosine production and regulation of renovascular tone [68].

### Intestinal AP activity and expression following CPB/DHCA

In this study, we found that exposure to CPB/DHCA resulted in increased ileal expression of iAP mRNA with a similar trend in the colon. We did not find similar changes in the jejunum, suggesting that watershed areas of the intestines may be more highly affected. The increase in mRNA expression was not associated with an increase in AP activity, indicating either concurrent loss of iAP protein or a delay between transcription and translation. Prior studies in murine models of ischemia-reperfusion and colitis demonstrate significant decreases in iAP activity with intestinal injury. Jejunal AP activity decreases after superior mesenteric artery/vein clamping [52, 53]. Decreased intestinal AP activity has also been shown in models of colitis [69] and necrotizing enterocolitis [70]. iAP loss is thought to increase susceptibility to injury and worsen intestinal barrier function, both of which are improved with interventions to increase intestinal AP activity [69–71]. Contrary to our findings, in both vascular clamping and colitis, loss of iAP activity was not balanced by an increase in iAP mRNA expression [53, 69]. Several possibilities could explain this difference, including severity of injury (prolonged vascular clamping compared to the more translationally relevant DHCA), location of sampling (jejunum vs ileum), and complexity of the injury model and the subsequent tissue response. Further studies are needed to better understand the mechanism and time course of these changes in intestinal AP as well as their relationship to injury severity.

### Renal AP activity and expression following CPB/DHCA

Our group has previously published that kidney tissue AP activity is significantly lower in piglets exposed to CPB/DHCA compared to anesthesia controls and that BiAP infusion increases renal AP activity [48]. Here we find that kidney AP mRNA expression is unaffected by CPB/DHCA, indicating that the decrease in renal tissue AP activity is secondary to loss of tissue AP without a concurrent increase in production. Both preclinical models and human studies have demonstrated loss of renal AP activity in a variety of pathologies including ischemia, interstitial nephritis, and obstructive uropathy [72–74]. Preclinical murine models of ischemia-reperfusion with therapeutic AP administration demonstrated improved histologic injury scores and increased cortical paO2, [75] potentially via increased adenosine signaling [28, 32, 76, 77]. To our knowledge, though, only one study has examined AP mRNA expression from kidney tissue. Contrary to our findings in CPB/DHCA, Kapojos et al found increased mesangial cell AP mRNA expression following TNFα stimulation in vitro [78]. The authors did not find similar increases in AP expression in other forms of kidney pathology such as nephritis and acute graft rejection. It is possible that upregulation of AP in the kidney is relatively unique to lipopolysaccharide/TNFα stimulation. Alternatively, the level of early cortical injury induced by CPB/DHCA may limit the ability of proximal tubule and glomerular cells to increase AP production until recovery has occurred.

### Lung AP activity and expression following CPB/DHCA

The lung was the only organ we studied where exposure to CPB/DHCA resulted in an increase in both AP activity and TNAP expression, a novel finding that has not been previously reported to our knowledge. Other disease models have used AP as a biomarker of lung injury, [79] but only recently have research efforts focused on understanding the importance of AP in lung pathology. TNAP lines the epithelial/mucosal surface of the lung with a predominance in the lower airways [50]. It is produced primarily by type 2 alveolar cells, although neutrophils may contribute [51]. Lipopolysaccharide and extracellular adenine nucleotides appear to be the most promising candidate targets of pulmonary epithelial AP under pathologic conditions. Intratracheal or intraperitoneal instillation of LPS increases neutrophil production of AP [80]. Ambroxol, a bronchial expectorant known to release AP-containing surfactant particles, increases lung tissue AP and leads to decreased pulmonary and serum LPS following intratracheal instillation of LPS [19]. Extracellular adenine nucleotides are released into the alveolar space during pulmonary infection or other pathologic conditions such as ventilator induced lung injury. There they act as danger-associated molecular patterns, leading to immune activation, capillary leak, and decreased surfactant production [81]. Low levels of extracellular adenine nucleotides in the alveoli are cleared sequentially by CD39 and CD73 to adenosine, but under pathologic conditions these enzymes become saturated [81] and TNAP becomes the primary enzyme responsible for clearance to adenosine [50]. Adenosine production in turn promotes anti-inflammatory signaling through pulmonary purinergic receptors and may also promote healing following acute lung injury [82]. Based on our findings, increased pulmonary production of AP may be part of the host response to the ischemia-reperfusion injury associated with CPB/DHCA. Further studies are required to confirm the primary cell type responsible for this increase in AP production and to understand the clinical importance of increased lung tissue AP.

### Effects of AP infusion on tissue AP activity

To our knowledge, changes in tissue-level AP activity following BiAP infusion have not previously been reported. Infusion of our highest dose of BiAP to piglets undergoing CPB/DHCA resulted in a significant increase of total AP activity in kidneys and liver only. Lower doses of AP did not significantly change tissue AP activity in any organ. BiAP infusion did not appear to substantially alter TNAP mRNA expression in any organ. Our high dose regimen used a similar bolus dosing to previous adult human studies (75 U/kg vs 67.5 U/kg) but a higher continuous infusion (25 U/kg/hr. vs 5.5–8.3 U/kg/hr), [38, 39, 83] which was needed to significantly raise circulating AP activity. While these human studies did not evaluate tissue-level changes, Peters et al did evaluate tissue distribution of a recombinant chimeric human AP molecule (human intestinal AP with the crown replaced by human placental AP) in adult minipigs [84]. Using iodine-125 labeled versions of this recombinant AP, the group demonstrated substantial delivery to the liver, supporting our findings of delivery of BiAP to the liver following CPB/DHCA. Contrary to our study, the authors did not find substantially more delivery to the kidneys compared to the other organs tested. It is possible that there are differences in the organ-specific delivery based on pediatric vs adult animals, infusion dose, disease type, or exact AP molecule administered. Alternatively, we cannot rule out the possibility that infusion of exogenous AP helps preserve native AP activity in the kidneys. This action could potentially occur through systemic dephosphorylation of toxic molecules resulting in less renal tubular epithelial exposure and subsequent decreased tubular epithelial injury. Continued studies are needed to clarify the exact tissue distribution of BiAP following CPB/DHCA.

### Limitations

Our study has several limitations. First is the small sample size. Swine models are useful for the study of complex physiology like cardiac surgery and CPB-induced organ injury [85] but have inherently greater variability than murine or cell based models. Therefore, it is possible that we were underpowered to detect more subtle differences in specific organs (type 2 error) that may have been identified with a larger sample size. To maintain study feasibility, we were only able to study a single, acute postoperative time point. Future studies should include serial time points throughout the window of postoperative critical illness (24–48 h) in order to determine ongoing changes in AP biology and their association with postoperative organ injury. In any animal model, there is the possibility that the metabolism of and physiologic response to a novel therapeutic may not adequately replicate similar responses in humans. We chose to use a porcine model due to the similarities in both the physiologic response to CPB and drug metabolism [86–91]. However, staged clinical trials in children and adults undergoing cardiac surgery are needed to definitively prove both the safety and efficacy of this therapy. While it is possible to differentiate among human AP isoenzymes/isoforms, [46] the previously published lack of available antibodies to exogenous BiAP and native non-human AP makes this differentiation challenging in animal models [70]. Therefore we cannot determine what proportion of changes in AP activity are directly due to BiAP delivery versus prevention of native AP depletion. Use of alternative measurement techniques such as mass spectrometry may be needed to measure changes in specific isoenzymes and to better track organ-specific drug delivery. Finally, this study was not designed to evaluate the impact of these tissue-level changes in AP. Future studies are warranted to evaluate the effects of native and exogenous AP on tissue inflammation and injury.

## Conclusions

Tissue AP activity varied significantly across the thoracoabdominal organs, with the highest activity found in the abdominal epithelial organs (kidney and intestines). The response to CPB/DHCA also varied among the organs studied. The kidneys demonstrated substantial loss of AP activity following CPB/DHCA with no evidence of reciprocal increase in AP expression, whereas the lungs showed increased AP activity likely resulting from increased AP expression. The ileum demonstrated a mixed picture (increased expression but no change in AP activity) indicating increased production coupled with ongoing enzyme loss. BiAP infusion primarily increased kidney and liver AP activity. Together, these findings suggest that the kidneys may be a primary candidate organ for BiAP therapy after CPB/DHCA with a decrease in native AP activity that is potentially correctable by BiAP infusion. Meanwhile the postoperative biology of intestinal and lung AP is complex and warrants additional mechanistic studies.

## Methods

### CPB/DHCA piglet model

The animal protocol was approved by the Institutional Animal Care and Use Committee of the University of Colorado Anschutz Medical Campus. The current study is a pre-specified secondary analysis of a protocol designed to assess the effects of BiAP infusion on post-CPB/DHCA kidney and intestinal injury. Piglets underwent CPB with DHCA as described in detail in our recent publication [48]. All CPB/DHCA animals underwent peripheral CPB through cannulation of the internal carotid artery and external jugular vein. We utilized a pediatric oxygenator (Sorin Group, USA), low volume tubing, and a standard roller pump under isoflurane anesthesia. The animals were cooled to 22 °C rectal temperature using the CPB circuit to induce circulatory arrest. CPB was stopped once the target temperature was reached and the animals remained in DHCA for 75 min. CPB was then restarted and the piglets were rewarmed to 36 °C rectal temperature in approximately 30 min. The animals were then separated from CPB and provided ICU care for 4 h including full mechanical ventilation with analgesic and inotropic support until euthanasia. Five CPB/DHCA animals received no BiAP infusion, while three groups of animals (*n* = 5 per group) received medical grade BiAP (bRESCAP, Alloksys, The Netherlands) administered as a bolus prior to initiation of CPB/DHCA followed by a continuous infusion until euthanasia in the following doses: low dose (3 U/kg bolus followed by 1 U/kg/hr. infusion); medium dose (15 U/kg bolus followed by 5 U/kg/hr. infusion); high dose (75 U/kg bolus followed by 25 U/kg/hr. infusion) [41, 65]. bRESCAP was chosen over other candidate AP molecules due to its favorable kinetics (short half-life allowing titration via continuous infusion) and its safety profile in adults undergoing CPB [41, 92]. An additional seven animals underwent general anesthesia with mechanical ventilation for 7 h without undergoing CPB/DHCA to serve as anesthesia-only controls. Organ samples for AP activity measurement and TNAP/iAP mRNA expression were collected at the time of euthanasia.

### Total AP activity in tissue

To determine total AP activity, 50 mg of tissue from kidney, liver, jejunum, ileum, colon, lungs (right apex and right lower lobe) and heart, which had been snap frozen in liquid N_2_ at the time of harvest, was homogenized in 500 μl of ALP assay buffer (Biovision, Milpitas, CA) then centrifuged at 11,000×g for 20 min. Supernatant was diluted (1:20 for kidney, jejunum; 1:10 for ileum, 1:5 for colon) or analyzed directly (liver, lungs, heart) using a commercially available DRI-CHEM analyzer (HESKA Lab Systems, Loveland, CO). This test quantifies AP activity through a standard colorimetric assay following cleavage of p-nitrophenyl phosphate, measuring total AP activity without differentiating among AP isoforms/isoenzymes.

### RNA preparation, RT, and real-time PCR

Total RNA was isolated from kidney, liver, jejunum, ileum, colon, lungs (right apex and right lower lobe) and heart tissue (snap frozen in liquid N_2_) using the RNeasy Mini Kit (Qiagen, Santa Clarita, CA) as described in the manufacturer’s protocol. RNA concentrations were quantified at 260 nm, and the purity and integrity were determined using a NanoDrop. RT and real-time PCR assays were performed to quantify steady-state mRNA levels of TNAP and iAP. cDNA was synthesized from 0.5 μg of total RNA. Custom made primers and probe were used for detection (Integrated DNA Technologies, Coralville, Iowa). TNAP: Primer 1: 5′-AGAAACCCTTCACTGCCATC-3′, Primer 2: 5′-GTAGTTGTCGTGCGCATAGT-3′, Probe: 5′−/56-FAM/TACAAGGTGGTGGGTGGTGAGAGA/36-TAMSp/− 3′. iAP: Primer 1: 5′-CACCTGTCTGTCCACGTTGT-3′, Primer 2: CTAAAGGGGCAGATGAATGG-3′, Probe: 5′−/56-FAM/CAATTCCCGTACCTGGCTCTGTCC/36-TAMSp/− 3′. Reporter dye emission was detected by an automated sequence detector combined with ABI Prism 7300 Real Time PCR System (Applied Biosystems, Foster City, CA). Real-time PCR quantification was performed with TaqMan b-actin controls and relative mRNA expression calculated using the 2^−ΔΔCT^ method.

### Statistics

Variable distribution was first tested using the Shapiro-Wilk test. Data were then expressed as mean (SD) or median (intra-quartile range) as appropriate for the distribution. One-way ANOVA or Kruskal-Wallis test were performed to assess for differences among groups. If a difference was identified among groups, subsequent post-hoc testing was performed using Student t-tests or non-parametric Mann-Whitney U-test to identify the differential groups. Due to the small number of groups compared, no correction was performed for multiple comparisons during post-hoc testing. A *p*-value < 0.05 was considered statistically significant. JMP Pro 14 (Cary, NC) and GraphPad Prism 6 (La Jolla, CA) were utilized for all statistical analyses.

## Data Availability

The datasets used and/or analyzed during the current study are available from the corresponding author on reasonable request.
